# Strength in numbers: effect of protein crowding on the shape of cell membranes

**DOI:** 10.1042/BST20210883

**Published:** 2022-10-10

**Authors:** Victoria Thusgaard Ruhoff, Guillermo Moreno-Pescador, Weria Pezeshkian, Poul Martin Bendix

**Affiliations:** 1 Niels Bohr Institute, University of Copenhagen, Blegdamsvej 17, 2100 Copenhagen, Denmark; 2 Niels Bohr International Academy, Niels Bohr Institute, University of Copenhagen, Blegdamsvej 17, 2100 Copenhagen, Denmark

**Keywords:** bending mechanism, entropic pressure, excluded volume, membrane curvature, membrane proteins, protein crowding

## Abstract

Continuous reshaping of the plasma membrane into pleomorphic shapes is critical for a plethora of cellular functions. How the cell carries out this enigmatic control of membrane remodeling has remained an active research field for decades and several molecular and biophysical mechanisms have shown to be involved in overcoming the energy barrier associated with membrane bending. The reported mechanisms behind membrane bending have been largely concerned with structural protein features, however, in the last decade, reports on the ability of densely packed proteins to bend membranes by protein–protein crowding, have challenged prevailing mechanistic views. Crowding has now been shown to generate spontaneous vesicle formation and tubular morphologies on cell- and model membranes, demonstrating crowding as a relevant player involved in the bending of membranes. Still, current research is largely based on unnatural overexpression of proteins in non-native domains, and together with efforts in modeling, this has led to questioning the *in vivo* impact of crowding. In this review, we examine this previously overlooked mechanism by summarizing recent advances in the understanding of protein–protein crowding and its prevalence in cellular membrane-shaping processes.

## Introduction

The conventional textbook presentation of a plasma membrane, containing individual proteins floating in a lipid bilayer, does not provide a realistic picture of the membrane system: a heterogeneous and dynamic environment comprising domains, protein clusters and a high degree of protein coverage. Historically, the plasma membrane was viewed as a fluid-mosaic bilayer with few proteins in a vast sea of lipids [1]. This simplified view has since been replaced by an elaborate model more firmly representing the proteins heterogeneously distributed in a lipid raft-containing bilayer, influenced by dynamic interactions with cellular components like the cytoskeleton [2–6]. With this appreciation of the complexity of the membrane in place, it is not surprising that investigating and determining the role of the plasma membrane in cellular processes is a challenging task, yet necessary for our continued understanding of how the plasma membrane controls some of the most essential activities of life.

The plasma membrane is the site of action for an abundance of cellular processes, many of which require membrane shape remodeling. Membrane bending is what allows for the uptake of nutrients, waste disposal, cell migration and much intra- and extracellular communication [7–10]. Central to membrane reshaping is efficient membrane bending facilitated by proteins. The plasma membrane naturally resists bending, due to the hydrophobic and hydrophilic forces governing its structure, as well as its inherent interactions with the cell cytoskeleton [11]. Spontaneous curvature of membranes can result from asymmetric distribution of lipids within the two leaflets [12, 13]. However, the distribution of the lipids that make up the plasma membrane, cannot alone explain the large number of distinct curvature-dependent processes cells maintain. Instead, certain proteins are responsible for driving curvature, by somehow providing the energy needed to break the barrier for bending. Advanced experimental techniques including super-resolution, fluorescent microscopy techniques and gene editing has allowed researchers to investigate the interrelations between protein structures and the mechanics of these membrane-shaping proteins at high resolution [14–16]. These investigations have shown that a variety of proteins have the ability to bend membranes via different mechanisms [17, 18]. In cells no single protein is orchestrating curvature alone, however, shared structural protein features have long been accepted as responsible drivers of membrane reshaping. These include the insertion of wedges into the bilayer in the form of amphipathic helices [19, 20], extracellular lectins through binding to glycolipids [21] or intrinsic curvature of membrane binding domains and protein scaffolding [22, 23].

**FIG. 1.**
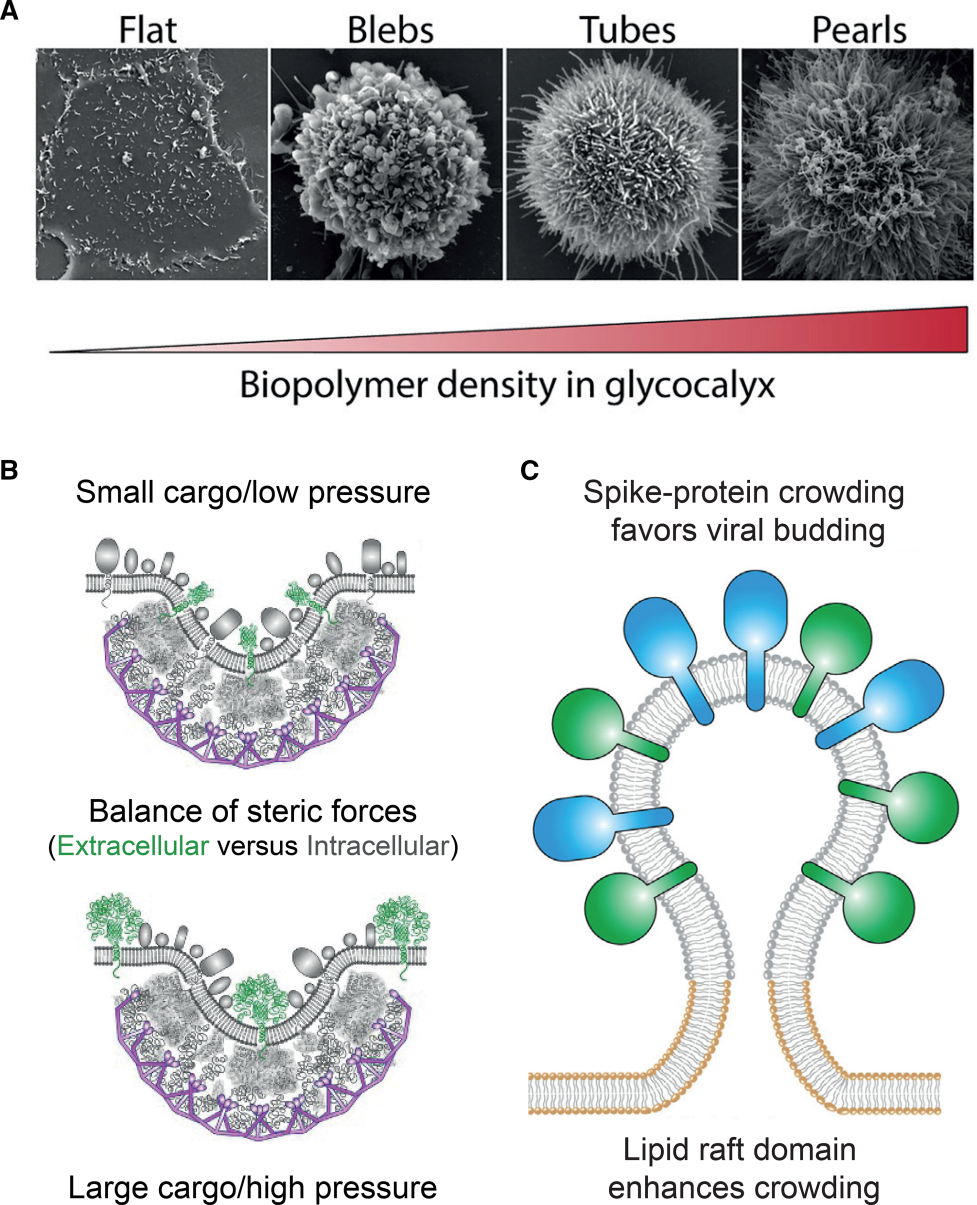
Examples of crowding in biological systems. (**A**) Crowding of mucin biopolymers on epithelial cell surfaces generate four distinct cell morphologies as a function of biopolymer denisty. From left to right the panel shows how increased crowding on the cell surface induces transitions in cell morphology from ‘flat’ to ‘pearled’. Reprinted from [26]. (**B**) Steric pressure amongst extracellular IDP domains modulates the protein composition within the endocytotic pit. As the membrane curvature is generated on the intracellular leaflet, the resulting negative curvature on the outer side causes size dependent sorting of extracellular proteins due to steric pressure. Figure adapted from [24]. (**C**) Tentative model of viral budding from a lipid raft domain (gray) due to crowding amongst the large ectodomains of the spikeproteins (blue and green). Figure adapted from [29].

Despite a large body of research highlighting conserved structural features as the driver for membrane bending, in the last decade an overlooked and highly disputed entropic mechanism, driving membrane bending via simple protein–protein crowding, has emerged, challenging prevailing views on how membranes adopt their shapes. Crowding drives curvature generation via lateral pressure created from stochastic collisions at one side of a membrane surface if that pressure is not counteracted on the opposing side of the membrane. This entropic mechanism is emerging as a relevant player in the dynamics of membrane-shaping machinery in cells including the generation of various cell surface morphologies, sorting of cargo in clathrin coated pits and potentially virus envelope budding (Figure 1B) [24–28].

In this review, we summarize recent literature to evaluate the impact of the crowding mechanism and its interplay with other structural mechanisms, e.g. wedge insertions and scaffolding. We comment on the biological relevance of crowding by examining the ability of the cell membrane to form crowded domains, and finally suggest directions for future quantitative experimental and modeling efforts needed to further our understanding of the role that protein–protein crowding plays in membrane remodeling.

## Asymmetric protein density affects membrane shape and bending

**FIG. 2.**
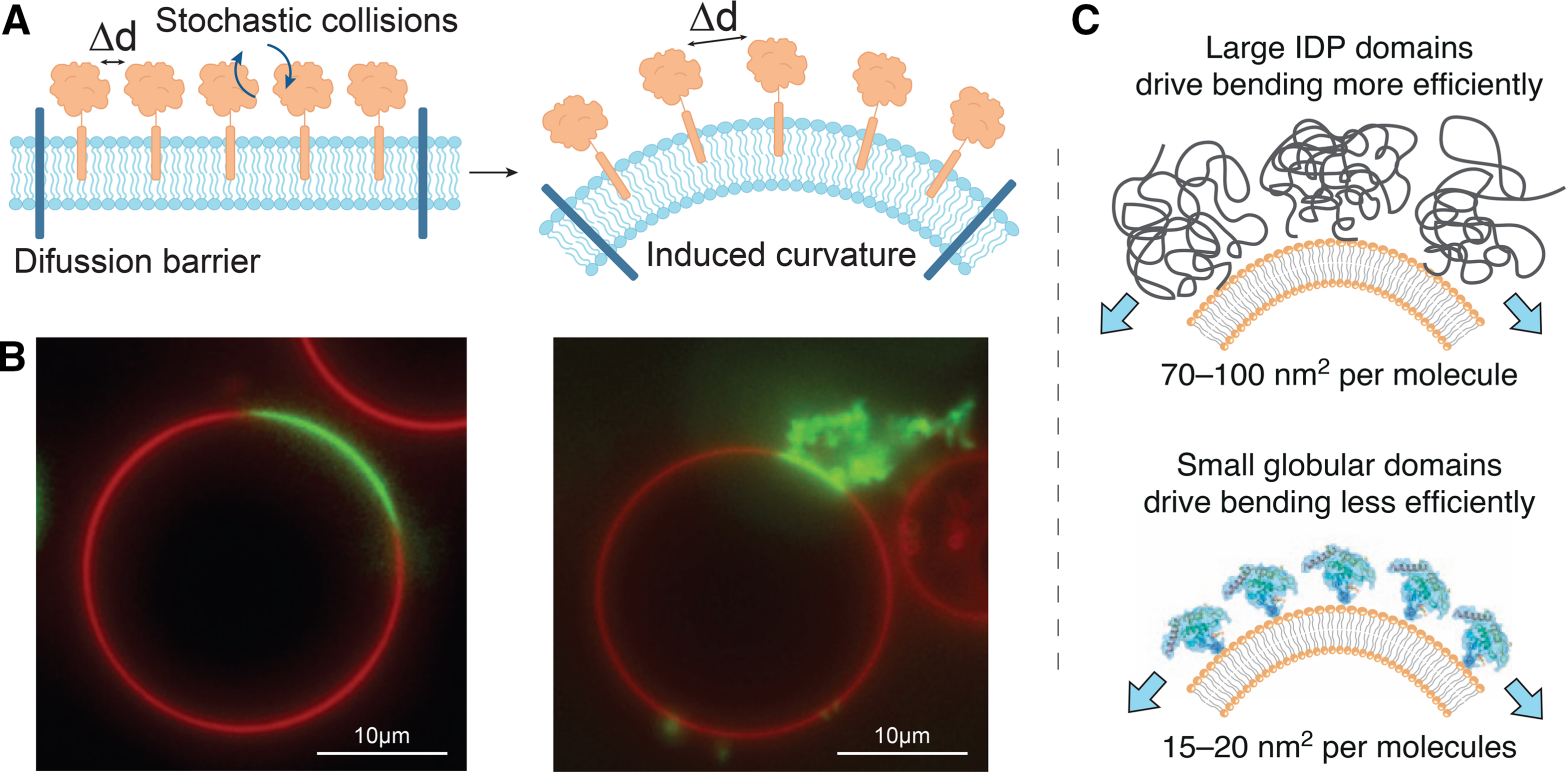
Bulky ecto- and IDP domains drive tubulation in model membranes. (**A**) Schematic illustrating how crowding between membrane-bound proteins (left panel) concentrated by a diffusion barrier can promote membrane bending, increasing Δd, to relieve the pressure (right panel). (**B**) Low (left panel) and high (right panel) density of Epsin1 ENTH domain in phase separated GUV membranes. Protein diffusion is limited by phase separation into lo (red) and ld (green) domains, resulting in spontaneous tubulation at high protein coverage in the ld domain (right panel). Reprinted from [25]. (**C**) Schematic depiction showing how IDP domains with a large hydrodynamic radius (top panel) creates membrane curvature more efficiently than smaller globular domains (lower panel). Reprinted from [24].

Various membrane-bound proteins continuously diffuse within the plasma membrane in a stochastic manner with frequent lateral collisions of their hydrophilic ectodomains in a narrow region above the membrane. The volume of this narrow region is controlled by the surface curvature and hence the translational entropy of the bound proteins will increase upon membrane bending. This gain of entropy will increase as the number of bound proteins increases, eventually overcoming the elastic energy penalty associated with bending. In other words, membrane bending increases the effective distance between the protein ectodomains (Figure 2A) and consequently lowers the chemical potential of the system. As a first approximation, the effect can be viewed as the buildup of pressure within a perfect gas, which is proportional to the volume and the number of bound molecules. As the concentration increases, the size of these molecules becomes important, and the excluded volume contribution must be considered [30]. If the concentration (protein coverage) continues to increase, there will be a regime where the proteins overlap and molecular interactions become important, which can cause strong membrane bending as seen e.g. for high densities of intrinsically disordered domains anchored to a membrane via BAR domains in Noguchi et al. [31].

With the heterogeneous complexity of the plasma membrane in mind, combined with estimated protein coverage of around 30–50% on the membrane surface [32, 33], it can be expected that non-specific crowding effects by proteins could play a role in biological processes involving membrane shaping. It was recently shown with a very intuitive, simple setup, that crowding of mucin biopolymers on a cell surface induces curvature, leading cells to form various morphologies dependent on the biopolymer density [26] (Figure 1A). Yet, identifying the effect from a single curvature-inducing mechanism in the complex environment of living cells is challenging, and thus the majority of experiments have been conducted in model membrane systems like giant unilamellar lipid vesicles (GUVs) (Figure 2B), where mechanisms can be readily isolated [24, 25, 34]. However, studies performed using a single type of protein incorporated in model membranes, having simple lipid compositions and no lipid leaflet asymmetry, can suffer from limited biological relevance. Other types of more biologically relevant model systems include giant plasma membrane vesicles (GPMVs) which are membranes isolated from cells. GPMVs preserve the complexity of the plasma membrane and importantly peripheral proteins preserve their inner or outer localization while integral membrane proteins retain their orientation [35–37]. Still, cell experiments provide the optimal foundation for investigating and verifying the biological relevance of membrane-shaping mechanisms.

Accumulated experimental results over the past decade have shown various protein domains are capable of inducing bending through crowding at certain threshold concentrations. Especially, attention has been on experiments concluding that proteins, generally not associated with membrane bending processes, can induce membrane curvature at high area coverage. An interesting example is GFP, a commonly used fluorescent protein, not containing any structural features associated with crowding, which has been shown to both induce spontaneous tubulation in GUVs and reduce vesicle size diameter in vesiculation experiments [14, 38]. In contrast with the notion that conserved structural features are orchestrating membrane bending, intrinsically disordered protein (IDP) domains, lacking complete 3D structure, have been identified as potential facilitators of membrane bending, via their comparably large hydrodynamic radii [24] (Figure 2C). Specifically, BAR proteins which have been thought to drive and stabilize membrane remodeling via a structural scaffolding mechanism, have recently been suggested by Snead et al. to be ‘potent drivers of membrane fission’ via crowding promoted by large IDP domains present in BAR domain containing proteins [22, 39, 40]. Recent simulations also showed that crescent-shaped BAR domains, linked to an IDP domain, induced both spherical and tubular shapes depending on the size of the IDP domain [31]. In addition, a novel method to validate the effect of crowding on membrane bending is to increase the protein volume by externally triggering unfolding of domains. Siaw et al. [41] demonstrated how steric pressure could be generated from chemically triggered protein unfolding, as proteins segregated into ordered lipid domains were shown to drive membrane deformation upon protein unfolding. Structural changes of integral proteins and membrane inserted domains are known to play a critical role in shaping membranes, but the work by Siaw et al. shows that it is relevant to account for conformational changes in the cytosolic or ectodomains when considering crowding mechanisms. This is specially important in biology as a great amount of cellular signaling pathway undergo some kind of conformational change.

The wide variety of structures and proteins associated with membranes and curvature-inducing events indicates that crowding is a mechanism with potential to influence a huge number of budding and fission processes in the cell. For example, viral budding of corona viruses or influenza viruses could partly be driven by protein crowding of various spike proteins containing large outer domains [42–44] (Figure 1C). In this context, an older theoretical study found the elastic constants of the membrane to change in the presence of anchored polymer chains on one side of the membrane. The bending rigidity of membranes was found to increase whereas the Gaussian rigidity was lowered due to the anchored polymers [45]. How this may affect budding viruses, which have saddle point curvatures before detachment from the plasma membrane, remains to be elucidated in future studies. Organelle membranes are also densely populated with proteins and exhibit highly curved regions with curvature radii that are comparable to the membrane thickness (∼10 nm) [46]. Yet, such high curvatures are often generated by oligomerization of curvature-inducing proteins, which in turn undermines the crowding effect of these proteins. Therefore, despite high protein densities, crowding may not have a significant effect in the formation of these membranes.

**FIG. 3.**
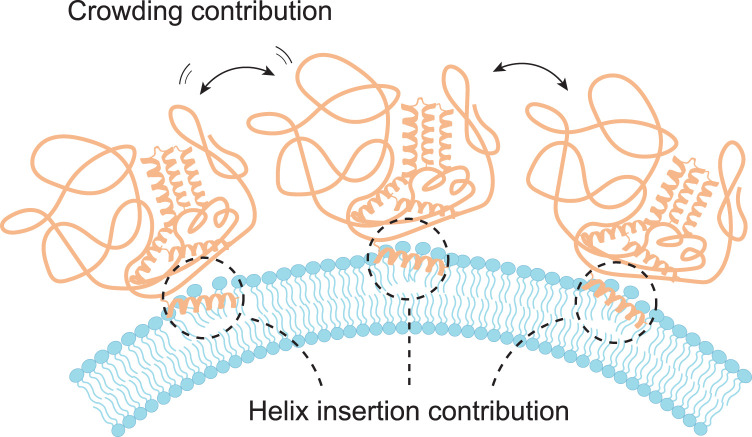
Curvature generation by dual crowding and wedge mechanism. Schematic illustration of membrane bending induced by a protein containing and IDP domain (crowding) and an amphipathic helix (wedge). Determining the driver of curvature generation *in vivo* is challenging as mechanisms such as helix insertion and crowding can work in synergy to orchestrate membrane bending.

Whether the crowding mechanism is biologically relevant, and underlying or assisting other bending mechanisms is still being debated. Especially the bending effect from helix insertion versus crowding has attracted significant attention, and although both mechanisms have been shown to induce curvature, disagreement still exists on the biological relevance and relative impact of each, since they can often be expected to work synergistically in the same process (Figure 3). Proteins have previously been suggested to induce curvature only with a certain amphipathic helix present in the construct, as upon mutations and modification, affecting for instance the insertion depth of these helices, membrane remodeling effects were altered [15, 47]. Intriguingly, it has subsequently been demonstrated that Epsin NH${\;}_{2}$-terminal homology (ENTH) domain, normally associated with membrane bending via wedge insertion, can actually induce spontaneous tubulation at crowding concentrations, even after deletion of its amphipathic helix [14, 25]. This observation, together with the fact that proteins have been shown to induce bending through crowding when the projected area reaches around 20% coverage, naturally suggests that crowding could provide an important contribution in facilitating membrane reshaping processes where the wedge mechanism has so far been seen as the single driver for membrane bending. For Epsin 1, the amphipathic helix is suggested to occupy at most 10% of the protein domain. Taken together with the measured size of the protein’s membrane footprint, the area occupied by the helix will then be on the order of 1% for physiologically relevant densities of ENTH domain or for the larger full-length Epsin 1 protein [25]. Considering that efficient membrane bending by helix insertions requires 10–30% area coverage occupied by the helix it is, unlikely that Epsin 1 generates curvature via wedge insertion alone [25]. However, work done by Kozlov et al. [48] on ENTH found contradicting results showing that ENTH without the amphipathic wedge was not able to form highly curved membrane structures as verified by cryo-EM. These experimental results were also backed-up by modeling showing that helix insertion was more efficient than crowding in bending membranes. Comparing these two studies we wish to emphasize two major points (i) the lipid mixtures used for the experimental assays in [25] contained a small fraction of the special lipid DPhPC (1,2-diphytanoyl-sn-glycero-3-phosphocholine), which is known to lower the threshold for membrane tubulation [38]. In ref. [48], however, another Folch lipid mix was used which is derived from natural membranes. (ii) The modeling performed in [48] compared two scenarios where the helix/protein area ratio was 0.1 and 0.3, respectively. Only the latter ratio showed negligible effects of crowding whereas a ratio of 0.1 showed significant synergistic effects of crowding and helix insertion in membrane bending. In this context, the helix/protein ratio for ENTH has been reported to be 0.1 ($A_{\mathrm{helix}}/A_{\mathrm{ENTH}}=1.6\;{\mathrm{nm}}^{2}/16\;{\mathrm{nm}}^{2}=0.1$) [25] and considering that the full length of the Epsin 1 protein would have an even larger projected membrane footprint, we can surmise that crowding could easily play a role for wild type Epsin’s ability to bend cellular membranes. It should in this context be emphasized that both modeling and experimental assays can be designed to reveal efficient tubulation by choosing specific experimental settings, or theoretical parameters, that favor tubulation by crowding or helix insertions. To avoid biased conclusions new investigations should rather focus on identifying the conditions for which crowding does play a role in the tubulation of membranes.

Cellular processes are unlikely to employ single mechanisms for curvature generation, but rather harness multiple of these mechanisms to remodel membrane shape. Literature highlights the difficulty in separating out single mechanisms for membrane bending due to the fact that curvature-generating proteins embody a multitude of features that are associated with membrane remodeling. For example, Amphiphysin1 contains intrinsic curvature and an IDP domain, both of which are thought to effectively crowd membranes, likewise Epsin 1 could drive curvature through insertion of its amphipathic helix and via its IDP domain [49]. Certain membrane fission events have previously been reported to be dependent on a balance between two otherwise curvature-generating mechanisms, as it was promoted by amphipathic helix insertion and simultaneously restricted by BAR scaffolding [15]. Even at dilute concentrations (where steric interactions are negligible) Steinkühler et al. [50] showed that spontaneous curvature is sufficient to induce fission events in GUVs. By controlling low densities of GFP proteins bound to the membranes of cell-sized lipid vesicles, curvatures could be generated comparable to those formed by BAR domain proteins. Naturally, synergistic effects between structured and stochastic mechanisms exists [48, 49, 51] and therefore, sophisticated approaches are required to disentangle these effects and resolve the mechanisms underlying membrane curvature generation. The taxing question then becomes whether these mechanisms are actually curvature sensing or curvature inducing, and if this difference can be measured through clever experimental design.

## Lateral confinement facilitates crowding

Relatively high protein coverage is needed for proteins to generate steric pressure in the 2D plane of the membrane sufficient to induce shape transitions [30]. However, local lateral confinement can assist in reaching the relevant protein densities necessary for bending a membrane [52]. In previously mentioned paper by Stachowiak et al. [25], an inhomogenous protein distribution is achieved in model membranes in the form of phase-separated GUVs containing liquid ordered (lo) and liquid disordered (ld) domains (Figure 2B). As the partitioning energy of proteins was different in different phases, each phase domain created a diffusion barrier, limiting the spread of the proteins over the entire membrane surface eventually causing enough pressure to overcome the threshold for membrane bending.

In cells, diffusion barriers consisting of membrane domains [53] or cortical actin network [54, 55], could be relevant in local and transient gathering of crowded domains needed for many small local processes such as clathrin-mediated endocytosis and viral budding. The formation of transient lipid raft domains has long been a suggested platform for the gathering of proteins, and the ability of different proteins to associate with these lipid ordered phases [56] has triggered a large interest for the biological implications of such domains. Experiments have identified some proteins to have affinity for either ordered or disordered phases in phase-separated GUV membranes [57, 58] and in isolated GPMVs [36, 59]. Although putative cell domains are most likely nanoscopic and transient, such work has served as evidence that the plasma membrane has an inherent ability to laterally organize the protein distribution in living cells. Along these lines controversial raft domains have long been thought to be the origin of processes like virus budding events [60]. Whether these domains have the ability to create sufficiently high protein–protein crowding remains unclear and has been challenged by recent work demonstrating that crowding opposes lipid phase separation [61]. This experiment, suggesting that the energetic contribution of crowding is high enough to disrupt membrane phases, highlights that lipid phase separation has obvious limitations when it comes to creating local enrichment of proteins. Yet, in [62] it was shown, in a meshless membrane model simulation, that densely anchored polymers can reduce line tension between lipid phases and thereby effectively stabilize microdomains. The effect was only verified for raft domains ≤100 nm. This work emphasizes the complexity of molecular interactions when considering the stability of microdomains and shows that lateral pressure from crowding and molecular effects on line tension can have opposite effects on the formation of small membrane domains.

In addition, as pointed out by Kozlov et al. [48], if upconcentration happens via protein interactive forces or oligomerization, then this inherently counteracts any crowding effect which is based on free diffusion and stochastic collisions. Therefore, the cell might need to employ more advanced machinery to crowd proteins at high surface densities. This can be achieved in many ways by the creation of diffusion barriers [6] as reviewed by Grinstein et al. [63]. Whether or not these can aid in protein crowding at the necessary concentration to break the energy barrier for bending remains to be investigated. Clustering of membrane proteins can also occur through direct and in-direct (membrane mediated) interactions, which creates a platform and seed for growth and bending to occur as suggested to be the role of the oligomerizing Matrix protein 1 involved in influenza virus budding [64, 65]. In general for proteins to generate membrane curvature via different mechanisms, clustering is essential and we refer to Johannes et al. [66] for further reading on this subject.

## Quantitative assessment of the crowding effect on membrane shape

Despite the evidence that crowding can act as a driver for membrane bending, both in cells and model membranes, the effect of crowding, and in which biological processes it plays a part, is still far from being fully elucidated. In the field, the focus has been on demonstrating crowding as a general mechanism, characterized by the spontaneous generation of membrane tubes, vesicles, pearls and similar structures [14, 24, 26]. Although the visual effect of crowding, on either a cell surface or a membrane system (GUV) can be clearly demonstrated, and relays a lot of useful information, new methods are needed which allow visualization of the bending process as it develops. This would in many cases require high resolution fluorescence imaging due to the high curvatures displayed by the biological processes relevant to crowding. Imaging the evolution of the shape changes is important to achieve a full mechanistic understanding of the crowding effect and in this context we note that methods used for characterizing the bending of membranes through percent of vesicles showing spontaneous tubulation, have been called into question by Sapp et al. [67] as they can be influenced by e.g. lipid heterogeneity effects.

**FIG. 4.**
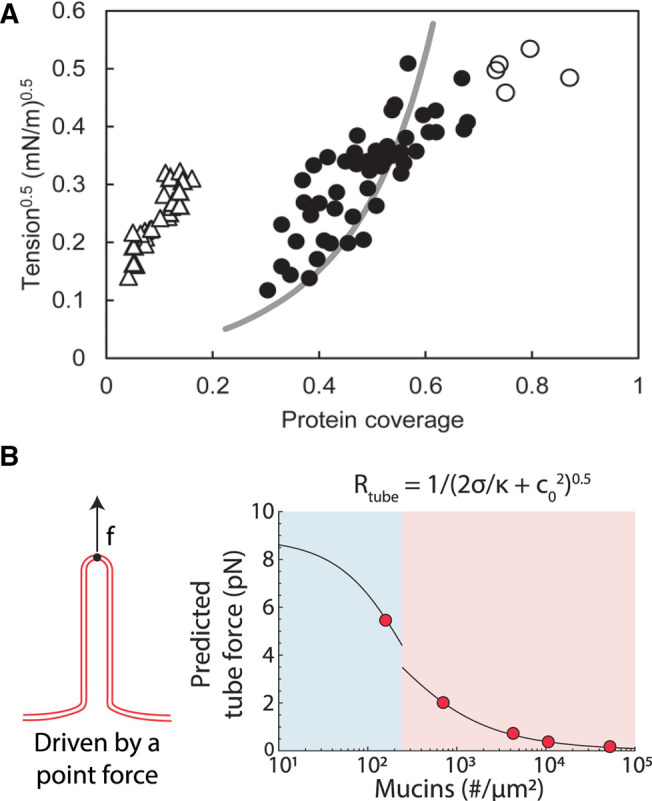
Quantitative assessment of curvature-generating mechanisms using micropipette aspiration. (**A**) Critical tension for which proteins induce shape instability in aspirated GUVs as a function of protein coverage. Endophilin BAR domains bind to the membrane via scaffolding and helix insertion (white triangles) whereas hisEGFP proteins bind to Ni-NTA lipids in the GUV and induce crowding with no helix insertion (black circles). The shaded line represents an instability theory based on repelling hard disks and empty circles represent high-density data for which the model breaks down. Using critical tension as a probe for membrane shape instability provides quantitative assessment of the crowding effect since higher tension counteracts bending. Reprinted from [34]. (**B**) Predicted tube force vs. the number of mucin biopolymers on a cell surface. The modeling reveals an expected decrease in the force needed to extract a tube from a crowded membrane as the protein density increases. Measuring tube force can serve as a measure to quantitatively assess the crowding effect. Reprinted from [26].

Designing experiments to identify the underlying bending mechanisms and provide quantitative evidence for crowding is difficult, thus modeling has been a key factor when discussing biologically relevant systems. However, disagreement on the significance of the modeled crowding effect still exists in published papers [48, 67], thus separating the crowding contribution from other mechanisms has proven challenging not only in living systems, but also in theoretical modeling of biological systems. All in all, more quantitative experimental evidence, along the lines presented by Chen et al. [34], is needed to support the modeling efforts. In this work, membrane bending or shape instability on the surface of GUVs was measured as a function of membrane tension, which was regulated by the micropipette aspiration of the GUVs. The threshold for nanoscale membrane bending on the surface of GUVs was indirectly quantified from the aspirated length at a given pipette pressure. The GUVs were immersed into solutions with various concentrations of either crowding proteins or scaffolding proteins to compare the relative effect. Their conclusion provide an indication that the crowding effect is weaker compared to the suggested combined helix and scaffolding effect of BAR domain proteins (Figure 4A).

If crowding plays a significant role in driving membrane reshaping, this potential ‘bending’ force should be measurable with highly sensitive force detection like e.g. optical tweezers which are routinely used for extracting membrane tethers from cells [68, 69], GPMVs [35, 36] and GUVs [70]. Shurer et al. [26] has presented modeling of the point force predicted from tube formations under the influence of crowding by mucins (Figure 4B). Mimicking vesicle and tube budding with ultra-sensitive tether extraction techniques, could, therefore, provide the means to quantitatively characterize the spontaneous curvature contribution from protein crowding and hence provide a more accurate measure for the contribution from crowding in bending membranes. Other methods capable of providing relevant measurements of the crowding effect, such as a recently developed probe for measuring the lateral membrane pressure from crowding via FRET [71] or fluorescence lifetime quantification [72], will provide a deeper understanding of the mechanistic effect of crowding. Quantitative measurements of crowding should also be exploited to assess the tunable bending effects available through molecular engineering or by adjusting solution conditions [73].

## Conclusion

Current knowledge obtained from multiple approaches including advanced experimental techniques and modeling has made it clear that crowding is indeed an important factor in shaping membranes at nanoscale. Looking forward, the focus must naturally shift towards identifying the processes in which crowding plays a role and characterize the relative, effective contribution of various biophysical membrane-shaping mechanisms. Advanced experimental techniques and rapid progresses in multiscale computer simulations now provide a fantastic opportunity to make a synergetic effort for decoupling and quantifying these contribution. In particular, the combination of highly sensitive force measuring tools like optical tweezers and quantitative imaging could reveal interesting details on how crowding differs for various proteins with different domain sizes and stalk lengths represented in cells. Future endeavors using a palette of experimental techniques together with modeling will undoubtedly give a more nuanced picture of the role of crowding in biology.

## Perspectives

- The plasma membrane of cells is a highly crowded environment containing numerous types of proteins which give rise to phenomena such as entropic pressure, excluded volume effects and steric repulsion among the proteins. Evidence is now emerging that these physical phenomena are responsible for bending membranes and hence could be critical factors in a plethora of essential cellular functions involving membrane remodeling. In combination with molecular engineering and model systems the crowding effect can be tuned to harness effective control over membrane shapes useful for biological reconstitution and for nanotechnology applications like drug delivery.
- Current thinking explains membrane shape modulations as driven by motors or by specific protein properties like amphiphilic helix insertion or scaffolding, but is challenged by the identification of protein–protein crowding as a driver of curvature.
- An increased focus on coherent modeling and experimental design to disentangle and quantify the contribution from crowding in membrane curvature generation is needed to fully validate the importance of this interesting physical effect.

## Abbreviations

ENTH: Epsin NH${\;}_{2}$-terminal homology
GUVs: giant unilamellar lipid vesicles
IDP: intrinsically disordered protein
GPMVs: giant plasma membrane vesicles
